# Author Correction: ATF3 induction prevents precocious activation of skeletal muscle stem cell by regulating H2B expression

**DOI:** 10.1038/s41467-023-41720-w

**Published:** 2023-09-25

**Authors:** Suyang Zhang, Feng Yang, Yile Huang, Liangqiang He, Yuying Li, Yi Ching Esther Wan, Yingzhe Ding, Kui Ming Chan, Ting Xie, Hao Sun, Huating Wang

**Affiliations:** 1 Department of Orthopaedics and Traumatology, Li Ka Shing Institute of Health Sciences, Chinese University of Hong Kong, Hong Kong SAR, China; 2 Center for Neuromusculoskeletal Restorative Medicine, Hong Kong Science Park, New Territories, Hong Kong SAR, China; 3 Department of Chemical Pathology, Li Ka Shing Institute of Health Sciences, Chinese University of Hong Kong, Hong Kong SAR, China; 4 Department of Biomedical Sciences, City University of Hong Kong, Hong Kong SAR, China; 5 Key Laboratory of Biochip Technology, Biotech and Health Centre, Shenzhen Research Institute of City University of Hong Kong, Shenzhen, 518172 China; 6 Division of Life Science, The Hong Kong University of Science and Technology, Hong Kong SAR, China

**Keywords:** Muscle stem cells, Regeneration, Quiescence

Correction to: *Nature Communications* 10.1038/s41467-023-40465-w, published online 17 August 2023

The original version of this Article contained an error in Fig. 2g, in which image for 5dpi Ctrl condition was inadvertently duplicated and the correct image for 5dpi iKO condition was originally omitted. This error has been corrected in both the PDF and HTML versions of the Article.

