# Supplementary material for: ATF3 induction prevents precocious activation of skeletal muscle stem cell by regulating H2B expression
Source: Nat Commun. 2023 Aug 17;14:4978. doi: 10.1038/s41467-023-40465-w (PMC10435463; doi:10.1038/s41467-023-40465-w)
Supplement: Supplementary file 1 — Supplementary Information [file 41467_2023_40465_MOESM1_ESM.pdf]

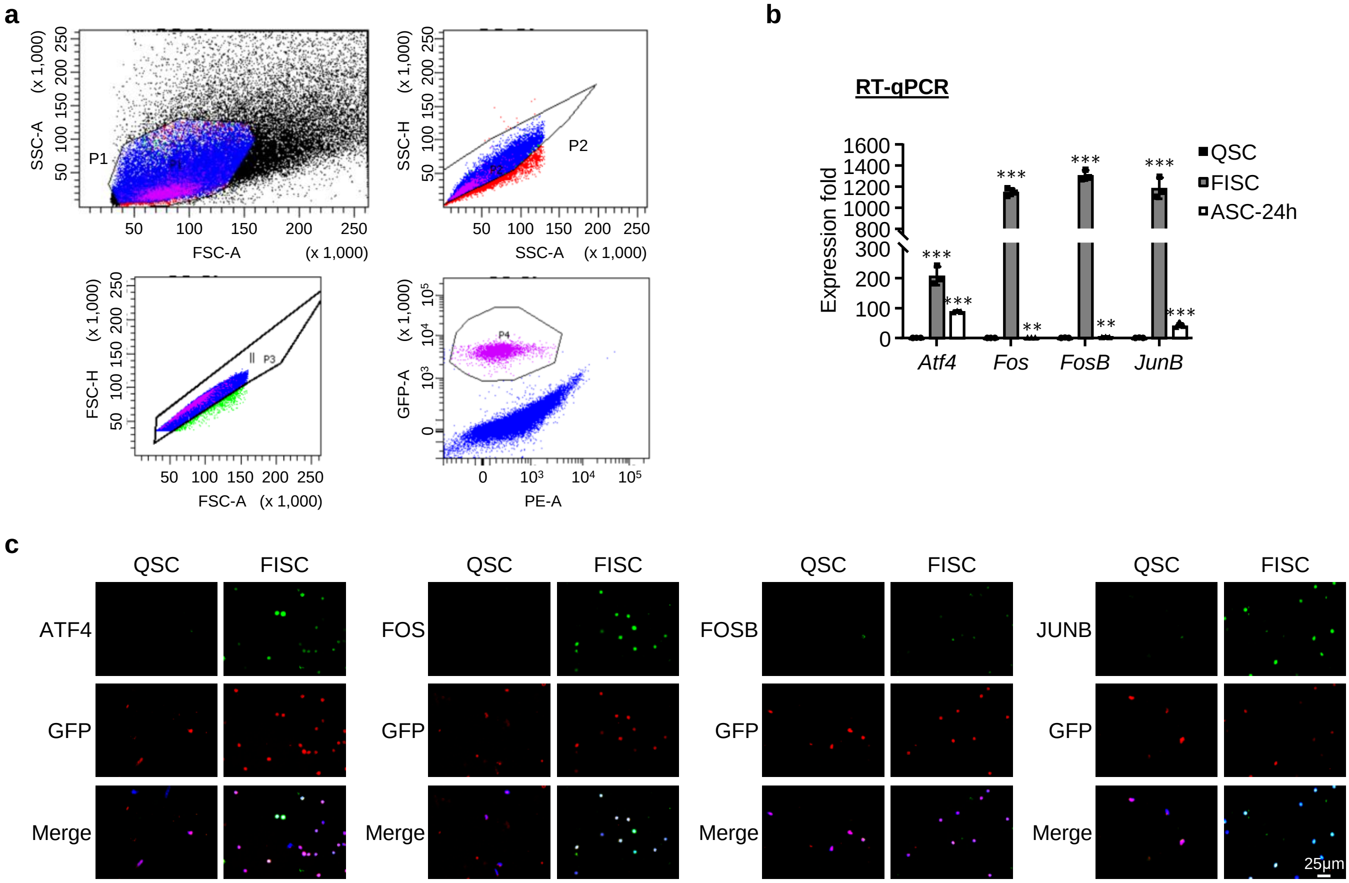

a
P1
SSC-A (x 1,000)
50 100 150 200 250
FSC-A (x 1,000)
P2
SSC-H (x 1,000)
SSC-A (x 1,000)
P3
FSC-H (x 1,000)
FSC-A (x 1,000)
P4
GFP-A (x 1,000)
PE-A
0 10³ 10⁴ 10⁵
b
RT-qPCR
Expression fold
0 100 200 300 800 1000 1200 1400 1600
QSC
FISC
ASC-24h
***
**
Atf4
Fos
FosB
JunB
c
QSC
FISC
ATF4
FOS
FOSB
JUNB
GFP
Merge
25μm

Supplementary Figure 1. *ATF3* is rapidly and transiently induced during SC early activation. a Sorting strategy for the isolation of SCs from Pax7 nGFP mice and *Atf3* Ctrl and iKO mice. P1-P4 was sequential gating and cells from P4 were collected as SCs. **b** qRT-PCR detection of *Atf4*, *Fos*, *Fosb* and *Junb* in QSC, FISC and ASC-24h from muscles of Tg: *Pax7-nGFP* mice. n = 3 mice per group. From left to right, *P* = 0.00034, 0.00000014, 0.00000064, 0.0018, 0.0000012, 0.0017, 0.000033 and 0.00019. **c** Immunofluorescence staining of ATF4, FOS, FOSB or JUNB and PAX7 proteins on QSCs and FISCs. Scale bar: 50μm. All the bar graphs are presented as mean ± SD., Student's t-test (two-tailed unpaired) was used to calculate the statistical significance (b): ∗∗p < 0.01, ∗∗∗p < 0.001. Source data are provided as a Source Data file.

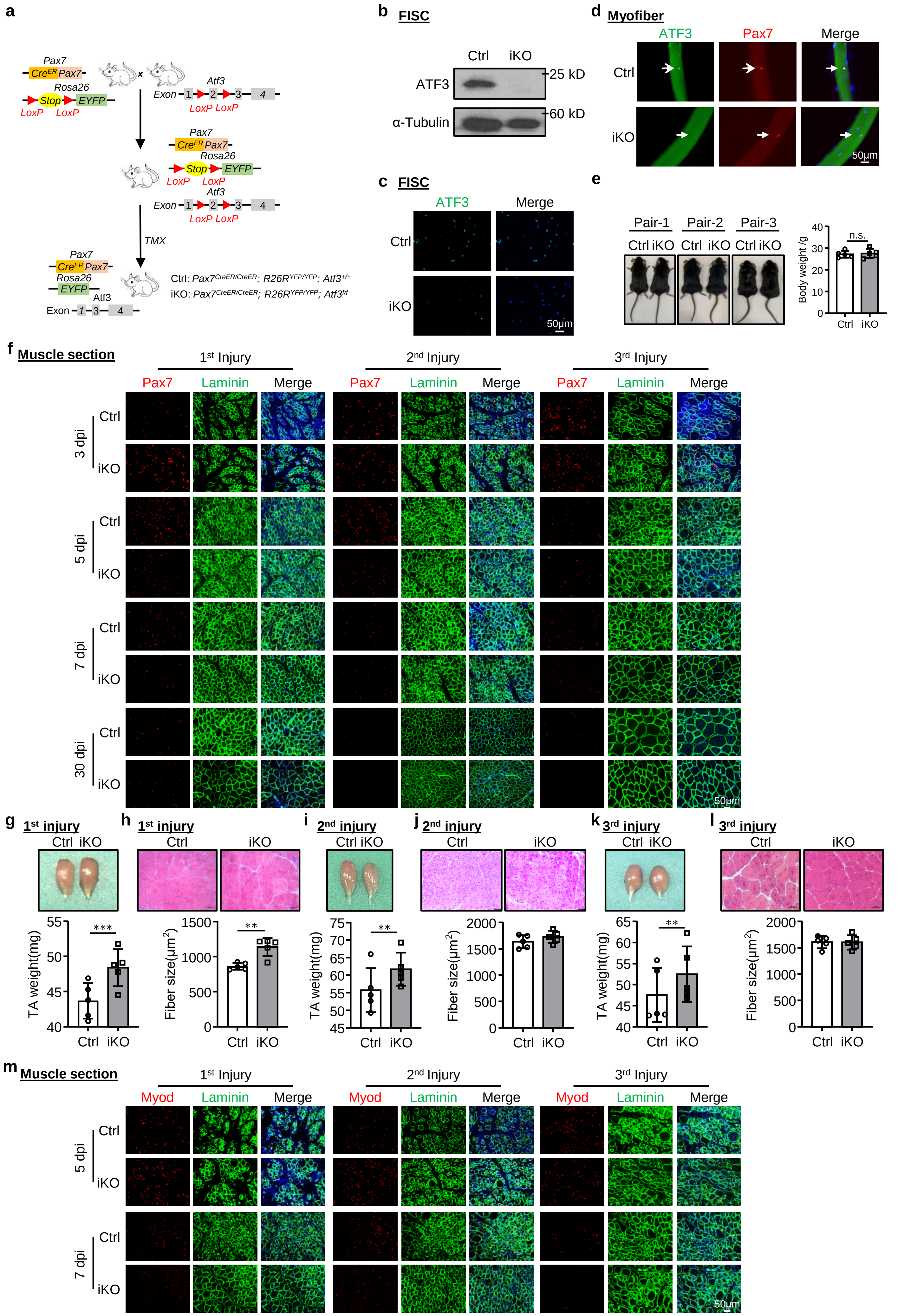

a
Pax7
Cre^ER Pax7
Rosa26
Stop
EYFP
LoxP LoxP
Atf3
Exon 1 2 3 4
TMX
Ctrl: Pax7^CreER/CreER; R26R^YFP/YFP; Atf3^+/+
iKO: Pax7^CreER/CreER; R26R^YFP/YFP; Atf3^f/f
b FISC
Ctrl iKO
ATF3
α-Tubulin
25 kD
60 kD
c FISC
ATF3 Merge
Ctrl
iKO
50μm
d Myofiber
ATF3 Pax7 Merge
Ctrl
iKO
50μm
e
Pair-1 Pair-2 Pair-3
Ctrl iKO Ctrl iKO Ctrl iKO
Body weight /g
n.s.
f Muscle section
1st Injury 2nd Injury 3rd Injury
Pax7 Laminin Merge
3 dpi
5 dpi
7 dpi
30 dpi
Ctrl
iKO
50μm
g 1st injury
h 1st injury
i 2nd injury
j 2nd injury
k 3rd injury
l 3rd injury
TA weight(mg)
Fiber size(μm²)
***
**
Ctrl iKO
m Muscle section
1st Injury 2nd Injury 3rd Injury
Myod Laminin Merge
5 dpi
7 dpi
Ctrl
iKO
50μm

Supplementary Figure 2. Short-term *Atf3* deletion accelerates acute injury-induced muscle regeneration. a Breeding scheme for generating inducible *Atf3* inducible conditional knock out (*Atf3* iKO) and control (Ctrl) littermates. **b** Loss of ATF3 protein in iKO FISC was confirmed by Western blot. α-Tubulin was used as a loading control. **c&d** IF staining for Pax7 and ATF3 on (c) FISCs or (d) single myofibers from Ctrl or *Atf3* iKO mice. Scale bar: 50μm. **e** No overt morphological difference or body weight change was observed in representative Ctrl vs iKO mice. *P* = 0.52. n = 5 mice per group. **f** IF staining of Pax7 (red), Laminin (green) and Dapi (blue) on Ctrl and iKO TA muscles on uninjured or 3, 5, 7, 30 days post the 1st, 2nd and 3rd round of injury. Scale bar: 50 μm. **g** TA muscles from Ctrl or *Atf3* iKO mice 30 days post the 1st round injury. n = 5 mice per group. *P* = 0.00073. **h** H&E staining of the above TA muscles. Scale bar: 50 μm. n = 5 mice per group. *P* = 0.0021. **i&j** TA muscles from Ctrl or *Atf3* iKO mice 30 days post the 2nd round injury. Scale bar: 50 μm (f). n = 5 mice per group. *P* = 0.0070 (e) and 0.17 (f). **k&l** TA muscles from Ctrl or *Atf3* iKO mice 30 days post the 3rd round injury. Scale bar: 50 μm (l). n = 5 mice per group. *P* = 0.0014 (k). and 0.94 (l). **m** IF staining of Myod (red) Laminin (green) and Dapi (blue) on Ctrl and iKO TA muscles at 3 and 5 days post the 1st, 2nd and 3rd round of injury. Scale bar: 50 μm; n = 5 mice per group for 3, and 5 days post 1st injury; n = 3 mice per group for 3, and 5 days post 2nd and 3rd injury. All the bar graphs are presented as mean ± SD., Student's t-test (two-tailed unpaired) was used to calculate the statistical significance (e, g-l): ∗p < 0.05, ∗∗p < 0.01, ∗∗∗p < 0.001. Source data are provided as a Source Data file.

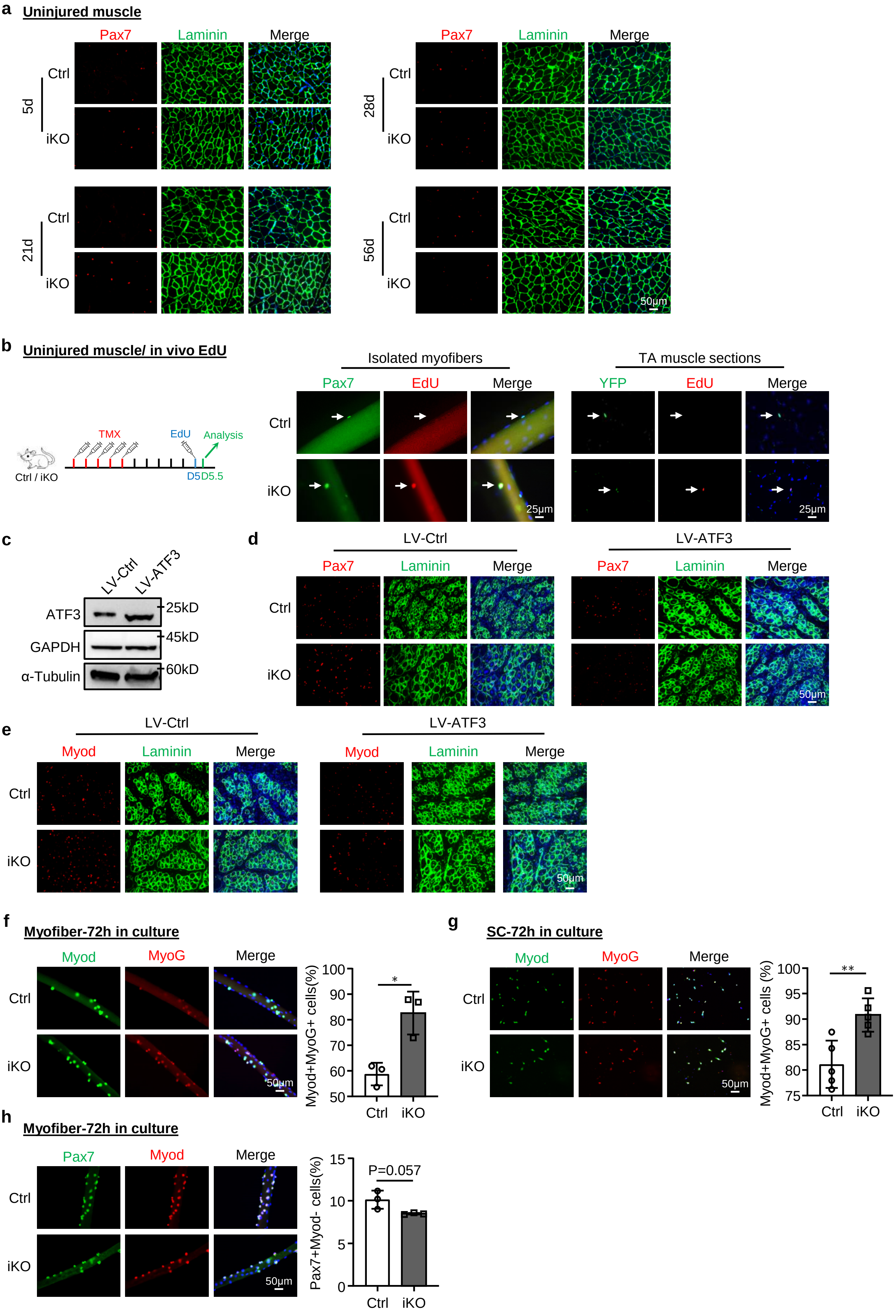
a
Uninjured muscle
Pax7
Laminin
Merge
Ctrl
iKO
5d
28d
21d
56d
50μm
b
Uninjured muscle/ in vivo EdU
TMX
EdU
Analysis
Ctrl / iKO
D5
D5.5
Isolated myofibers
TA muscle sections
Pax7
EdU
Merge
YFP
Ctrl
iKO
25μm
c
LV-Ctrl
LV-ATF3
ATF3
GAPDH
α-Tubulin
25kD
45kD
60kD
d
LV-Ctrl
LV-ATF3
Pax7
Laminin
Merge
Ctrl
iKO
50μm
e
LV-Ctrl
LV-ATF3
Myod
Laminin
Merge
Ctrl
iKO
50μm
f
Myofiber-72h in culture
Myod
MyoG
Merge
Ctrl
iKO
50μm
Myod+MyoG+ cells(%)
100
90
80
70
60
50
*
Ctrl
iKO
g
SC-72h in culture
Myod
MyoG
Merge
Ctrl
iKO
50μm
Myod+MyoG+ cells (%)
100
95
90
85
80
75
**
Ctrl
iKO
h
Myofiber-72h in culture
Pax7
Myod
Merge
Ctrl
iKO
50μm
Pax7+Myod- cells(%)
15
10
5
0
P=0.057
Ctrl
iKO

Supplementary Figure 3. *Atf3* deletion provokes SC premature activation and pseudo-regeneration in homeostatic muscle. a IF staining of Pax7 (red) Laminin (green) and Dapi (blue) on uninjured Ctrl or iKO muscles 5, 21, 28 and 56 days after TMX injection. Scale bar: 50 µm. **b** Left: Schematic outline of the EdU assay performed in the study. Middle: EdU (red) and Pax7 (green) staining on freshly single myofibers from the above injected muscles; right: EdU (red) and YFP (green) staining on the above muscles 12h after EdU injection. Scale bar: 25 µm; n = 3 mice per group. **c** C2C12 cells were infected with an ATF3 expressing lentivirus and the ATF3 over-expression was confirmed by Western blotting. α-Tubulin and GAPDH were used as loading controls. **d** ATF3 expressing lentivirus was injected into Ctrl or iKO TA muscles 1 day after $BaCl_2$ injury. IF staining of Pax7 (red) and Laminin (green) was performed on the TA muscles collected at 5 days after infection. Scale bar: 50 µm. n = 4 mice per group. **e** IF staining of Myod (red) and Laminin (green) was performed on the above TA muscles. Scale bar: 50 µm. n = 4 mice per group. **f** Left: Single myofibers were isolated from Ctrl or iKO mice and cultured for 72h before IF staining of Myod (green) and MyoG (red) was performed. Scale bar: 50 µm. Right: Quantification of the percentage of the Myod+MyoG+ SCs. n = 3 mice per group. P = 0.012. **g** Left: IF staining of Myod (green) and MyoG (red) on SCs isolated from Ctrl or iKO mice and cultured for 72h. Scale bar: 50 µm. Right: Quantification of the percentage of Myod+/MyoG+ SCs on single myofibers; n = 3 mice per group. P = 0.0050. **h** Left: IF staining of Pax7 (green) and Myod (red) on the above single myofibers. Scale bar: 50 µm. Right: Quantification of the percentage of Pax7+Myod+ SCs. n = 3 mice per group. P = 0.057. All the bar graphs are presented as mean ± SD., Student's t-test (two-tailed unpaired) was used to calculate the statistical significance (f-h): $*p < 0.05$, $**p < 0.01$. Source data are provided as a Source Data file.

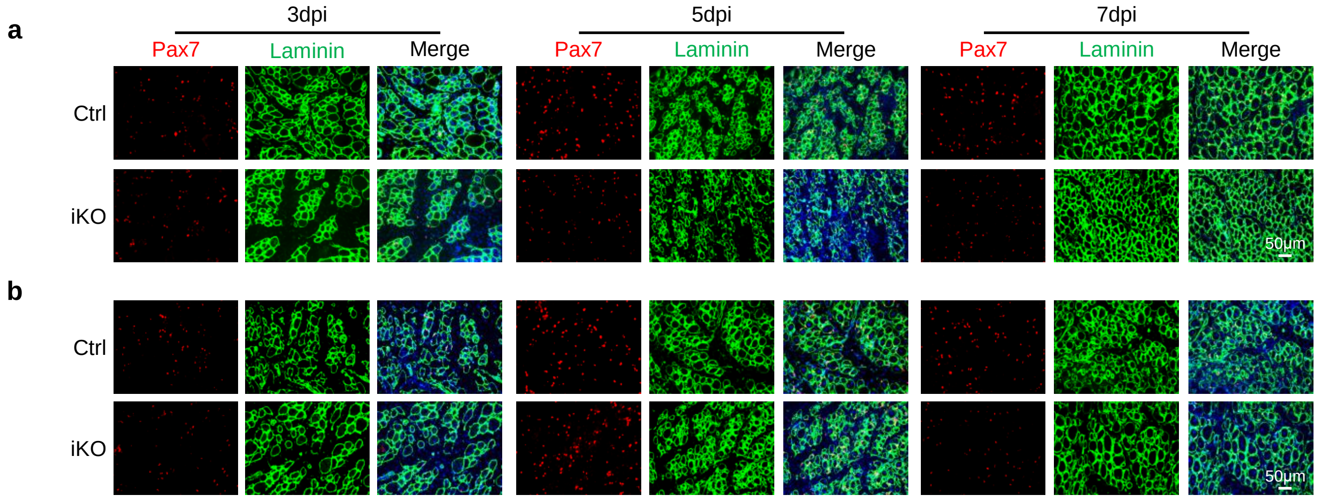
a
3dpi
5dpi
7dpi
Pax7
Laminin
Merge
Ctrl
iKO
50μm
b
Ctrl
iKO
50μm

Supplementary Figure 4. Long-term *Atf3* deficiency in *Atf3* iKO mice depletes SC pool and impairs muscle regeneration. a 30 days chasing after TMX injection was given before $BaCl_2$ injection. IF staining of Pax7 (red) and Laminin (green) was performed on the TA muscles collected at 3, 5 and 7 dpi after the 30 days chasing period. Scale bar: 50 μm. n = 5 mice per group. **b** 120 days chasing after TMX injection was given before $BaCl_2$ injection. IF staining of Pax7 (red) and Laminin (green) was performed on the TA muscles collected at 3, 5 and 7 dpi after the 120 days chasing period. Scale bar: 50 μm. n = 5 mice per group.

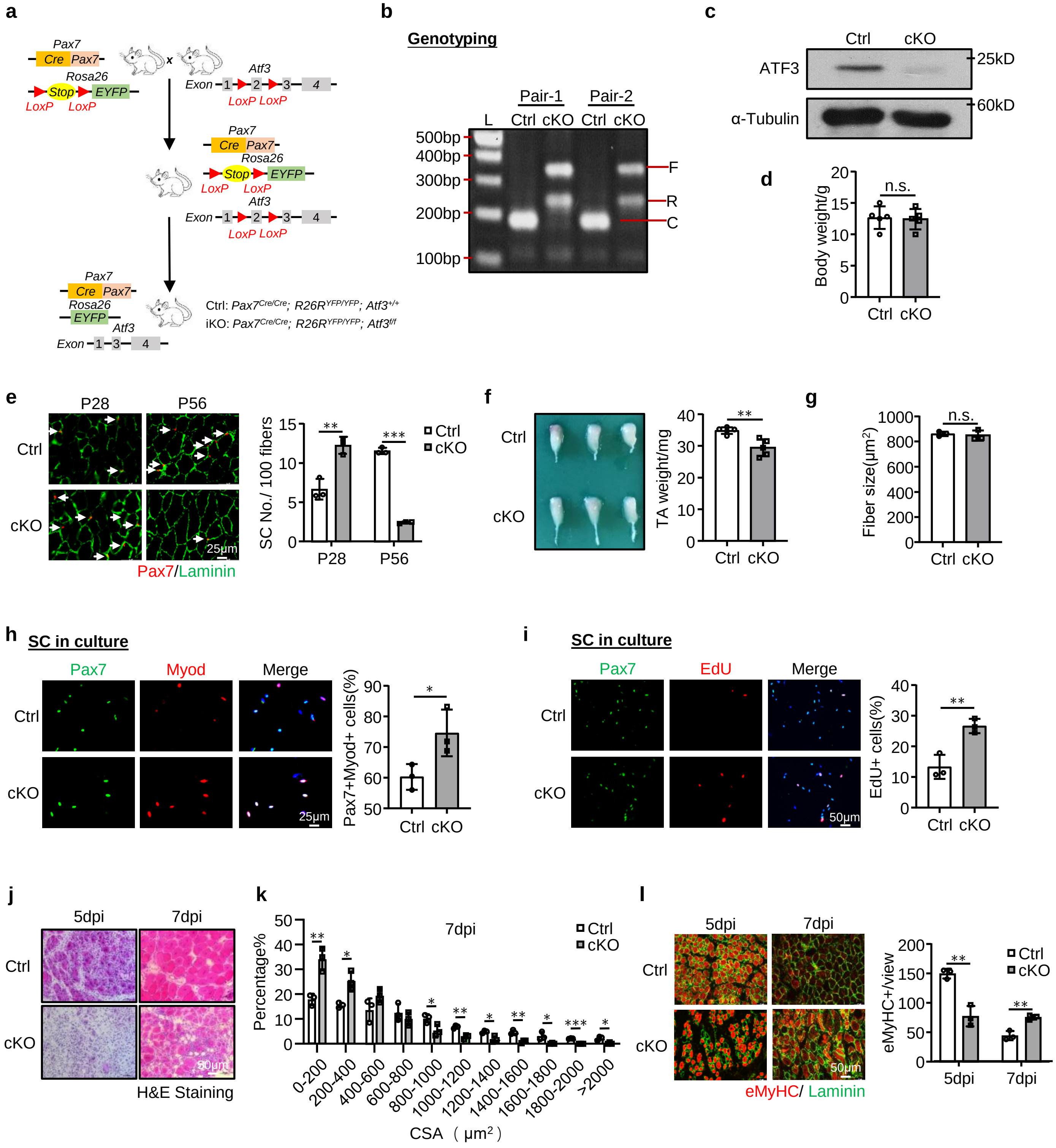

a
Pax7
Cre Pax7
Rosa26
Stop EYFP
LoxP LoxP
Atf3
Exon 1 2 3 4
LoxP LoxP
Ctrl: Pax7Cre/Cre; R26RYFP/YFP; Atf3+/+
iKO: Pax7Cre/Cre; R26RYFP/YFP; Atf3f/f
b
Genotyping
Pair-1 Pair-2
L Ctrl cKO Ctrl cKO
500bp
400bp
300bp
200bp
100bp
F
R
C
c
Ctrl cKO
ATF3
α-Tubulin
25kD
60kD
d
Body weight/g
n.s.
Ctrl cKO
e
P28 P56
Ctrl
cKO
25μm
Pax7/Laminin
SC No./ 100 fibers
Ctrl
cKO
P28 P56
f
Ctrl
cKO
TA weight/mg
g
Fiber size(μm²)
n.s.
h
SC in culture
Pax7 Myod Merge
25μm
Pax7+Myod+ cells(%)
i
SC in culture
Pax7 EdU Merge
50μm
EdU+ cells(%)
j
5dpi 7dpi
50μm
H&E Staining
k
7dpi
Percentage%
0-200 200-400 400-600 600-800 800-1000 1000-1200 1200-1400 1400-1600 1600-1800 1800-2000 >2000
CSA (μm²)
l
5dpi 7dpi
50μm
eMyHC/ Laminin
eMyHC+/view

Supplementary Figure 5. Long-term *Atf3* deficiency in *Atf3* cKO mice depletes SC pool and impairs muscle regeneration. **a** Breeding scheme for generating inducible *Atf3* conditional knock out (*Atf3* cKO) and control (Ctrl) littermates. **b** Genotyping results of the Ctrl and cKO mice. (F, *Atf3* Floxed; R, Cre recombinase in the *Pax7* locus; C, WT Ctrl). **c** Loss of ATF3 protein in the cKO FISCs was confirmed by western blot. α-Tubulin was used as a loading control. **d** No overt body weight change was observed in Ctrl vs cKO mice at postnatal 28 days. P = 0.83. **e** IF staining of Pax7 (red) and Laminin (green) at p28 and p56 on uninjured TA muscles from the Ctrl or cKO mice. Scale bar: 50 µm. P = 0.0046 and 0.0000030. **f** Decreased TA muscle size and weight were observed in cKO mice at p28p. P = 0.0028. **g** Quantification of the average CSAs of fibers at p28 ; P = 0.63. **h** IF staining of Pax7 (green) and MyoD (red) on SCs from the Ctrl or cKO mice after cultured for 24 h. Scale bar: 25 µm. P = 0.046. **i** from Ctrl or cKO mice were cultured for 24 h before treating with EdU for 6 h and staining for EdU (red) and Pax7 (green). Scale bar: 50 µm. P = 0.0071. **j** H&E staining of the TA muscles collected at 5 and 7 dpi from Ctrl or cKO mice. Scale bar: 50 µm. **k** CSAs of newly formed fibers were quantified from the above-collected TA muscle at 7dpi and the distribution is shown. From left to right, P = 0.0055, 0.013, 0.043, 0.0026, 0.020, 0.0038, 0.030, 0.0000085 and 0.018. **l** IF staining of eMyHC (red) and laminin (green) was performed on the above TA muscles collected at 5 and 7 dpi. Scale bar: 50 µm. P = 0.0030 and 0.0038. n=5 mice per group (d, f); n=3 mice per group (e, g-l). All the bar graphs are presented as mean ± SD., Student's t test (two-tailed unpaired) was used to calculate the statistical significance (f-k, m-n): $*p < 0.05$, $**p < 0.01$, $***p < 0.001$. n.s., no significance. Source data are provided as a Source Data file.

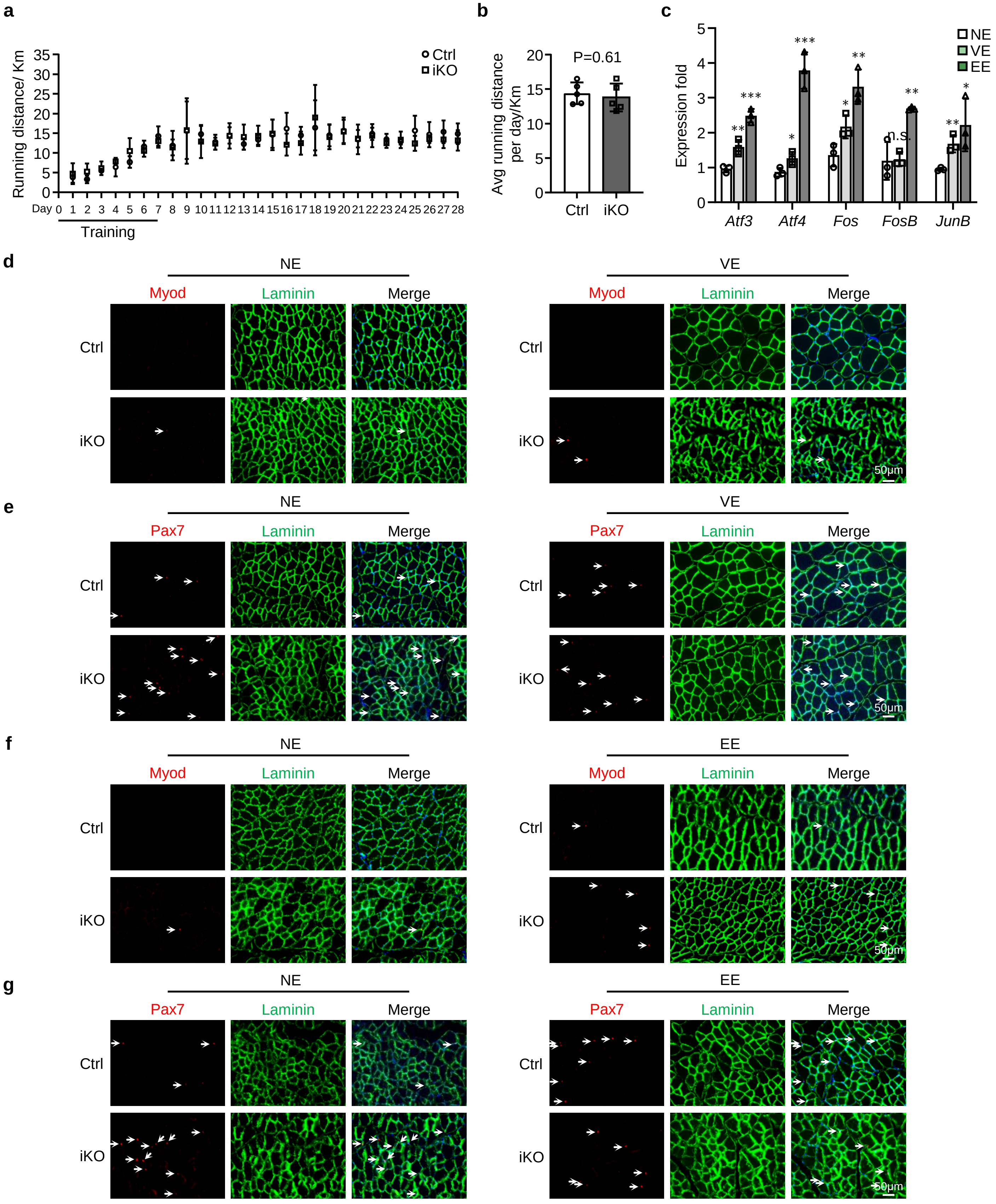

a
Running distance/ Km
Ctrl
iKO
Day 0 1 2 3 4 5 6 7 8 9 10 11 12 13 14 15 16 17 18 19 20 21 22 23 24 25 26 27 28
Training
b
Avg running distance per day/Km
P=0.61
Ctrl
iKO
c
Expression fold
NE
VE
EE
Atf3
Atf4
Fos
FosB
JunB
n.s.
d
NE
VE
Myod
Laminin
Merge
Ctrl
iKO
50μm
e
Pax7
f
EE
g

Supplementary Figure 6. ***Atf3*** **deletion induces SC activation during voluntary and endurance exercises. a** Ctrl and iKO mice were subject to voluntary running wheels for 1 week and followed by 3 more weeks of running. The daily running distance of Ctrl and iKO mice was recorded. n = 5 mice per group. **b** The average running distance per day of Ctrl and iKO mice. n = 5 mice per group. *P* = 0.61. **c** qRT-PCR detection of *Atf3*, *Atf4*, *Fos*, *FosB* and *Junb* in FISC isolated from Ctrl muscles NE, VE or EE. n = 3 mice per group. From left to right, *P* = 0.0091, 0.00026, 0.035, 0.00073, 0.038, 0.0044, 0.89, 0.0090, 0.0076 and 0.043 **d** IF staining of Myod (red) and Laminin (green) was performed on the TA muscles collected from Ctrl or iKO mice with VE or Ctrl with no exercise (NE). Scale bar: 50 µm. **e** IF staining of Pax7 (red) and Laminin (green) on the above collected TA muscles Scale bar: 50 µm. **f** IF staining of Myod (red) and Laminin (green) on the TA muscles collected from the Ctrl and iKO mice with EE or Ctrl with no exercise (NE). Scale bar: 50 µm. **g** IF staining of Pax7 (red) and Laminin (green) on the above collected TA muscles. Scale bar: 50 µm. All the bar graphs are presented as mean ± SD., Student's t-test (two-tailed unpaired) was used to calculate the statistical significance (b-c): ∗p < 0.05, ∗∗p < 0.01, ∗∗∗p < 0.001. n.s., no significance. Source data are provided as a Source Data file.

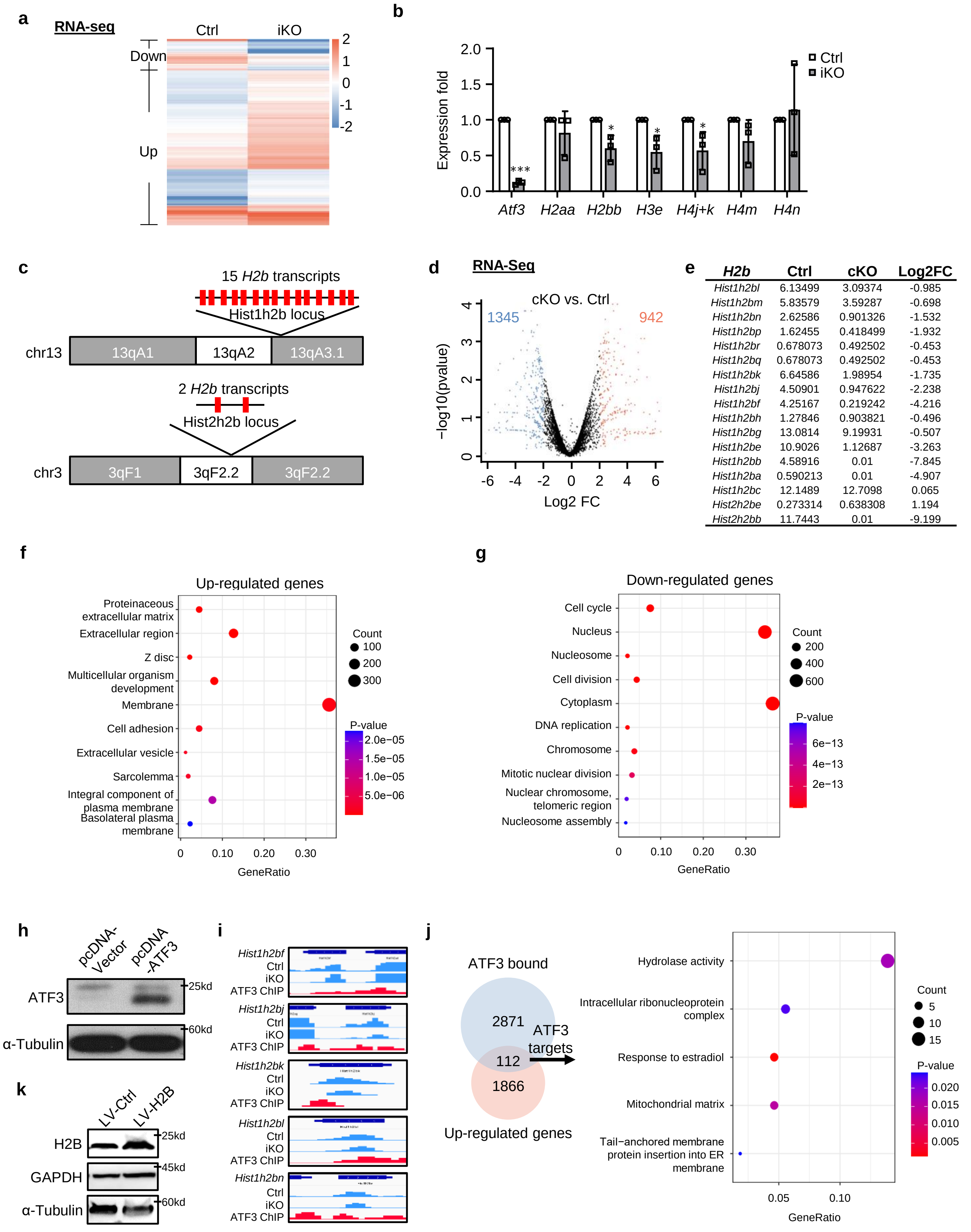

H2b	Ctrl	cKO	Log2FC
Hist1h2bl	6.13499	3.09374	-0.985
Hist1h2bm	5.83579	3.59287	-0.698
Hist1h2bn	2.62586	0.901326	-1.532
Hist1h2bp	1.62455	0.418499	-1.932
Hist1h2br	0.678073	0.492502	-0.453
Hist1h2bq	0.678073	0.492502	-0.453
Hist1h2bk	6.64586	1.98954	-1.735
Hist1h2bj	4.50901	0.947622	-2.238
Hist1h2bf	4.25167	0.219242	-4.216
Hist1h2bh	1.27846	0.903821	-0.496
Hist1h2bg	13.0814	9.19931	-0.507
Hist1h2be	10.9026	1.12687	-3.263
Hist1h2bb	4.58916	0.01	-7.845
Hist1h2ba	0.590213	0.01	-4.907
Hist1h2bc	12.1489	12.7098	0.065
Hist2h2be	0.273314	0.638308	1.194
Hist2h2bb	11.7443	0.01	-9.199

Supplementary Figure 7. ATF3 regulates *H2B* gene expression and nucleosome patterning. a RNA-Seq was performed in FISCs from the Ctrl and iKO cells and heat maps indicating differential gene expression levels (Log2[FPKM]) in the *Atf3* iKO vs. Ctrl. **b** RT–qPCR validation of the expression of selected histone genes in the iKO vs. Ctrl. From left to right, *P* = 0.00000079, 0.018, 0.028 and 0.047. **c** Illustration of the 2 clusters of Histone H2B coding genes on mouse chr3 and chr13. **d** RNA-Seq was performed in FISCs from the Ctrl and cKO mice. DEGs were identified in the cKO vs. Ctrl. **e** FPKM and Log2 fold change (FC) of *H2b* genes from the above RNA-Seq. **f&g** Gene ontology (GO) analyses of the above identified up and down-regulated genes in D. The top 10 enriched GO terms ranked by gene ratio (proportion of genes annotated for each GO term) are shown. Dots are colored by adjusted P value (fold change) and their size corresponds to the gene counts annotated to each GO term. **h** ATF3 was overexpressed in C2C12 with a pcDNA3.1-ATF3 plasmid. α-Tubulin was used as a loading control. **i** Genomic snapshots of 5 of the above identified *H2b* genes (*Hist1h2bf*, *Hist1h2bj*, *Hist1h2bk*, *Hist1h2l* and *Hist1h2bn*) with ATF3 binding in their TSSs (ChIP-Seq tracks) and down-regulated by *Atf3* deletion (RNA-Seq tracks). **j** Left: Venn diagrams show the overlapping (112 genes) between the ATF3 ChIP-Seq target genes (2871) and the up-regulated genes (1866). Right: GO analysis of the above 112 genes. **k** C2C12 cells were infected with a H2B expressing lentivirus and the H2B overexpression is confirmed by Western blotting. α-Tubulin and GAPDH were used as loading controls. All the bar graphs are presented as mean ± SD., Student's t-test (two-tailed unpaired) was used to calculate the statistical significance (b): $*p < 0.05$, $***p < 0.001$. Source data are provided as a Source Data file.

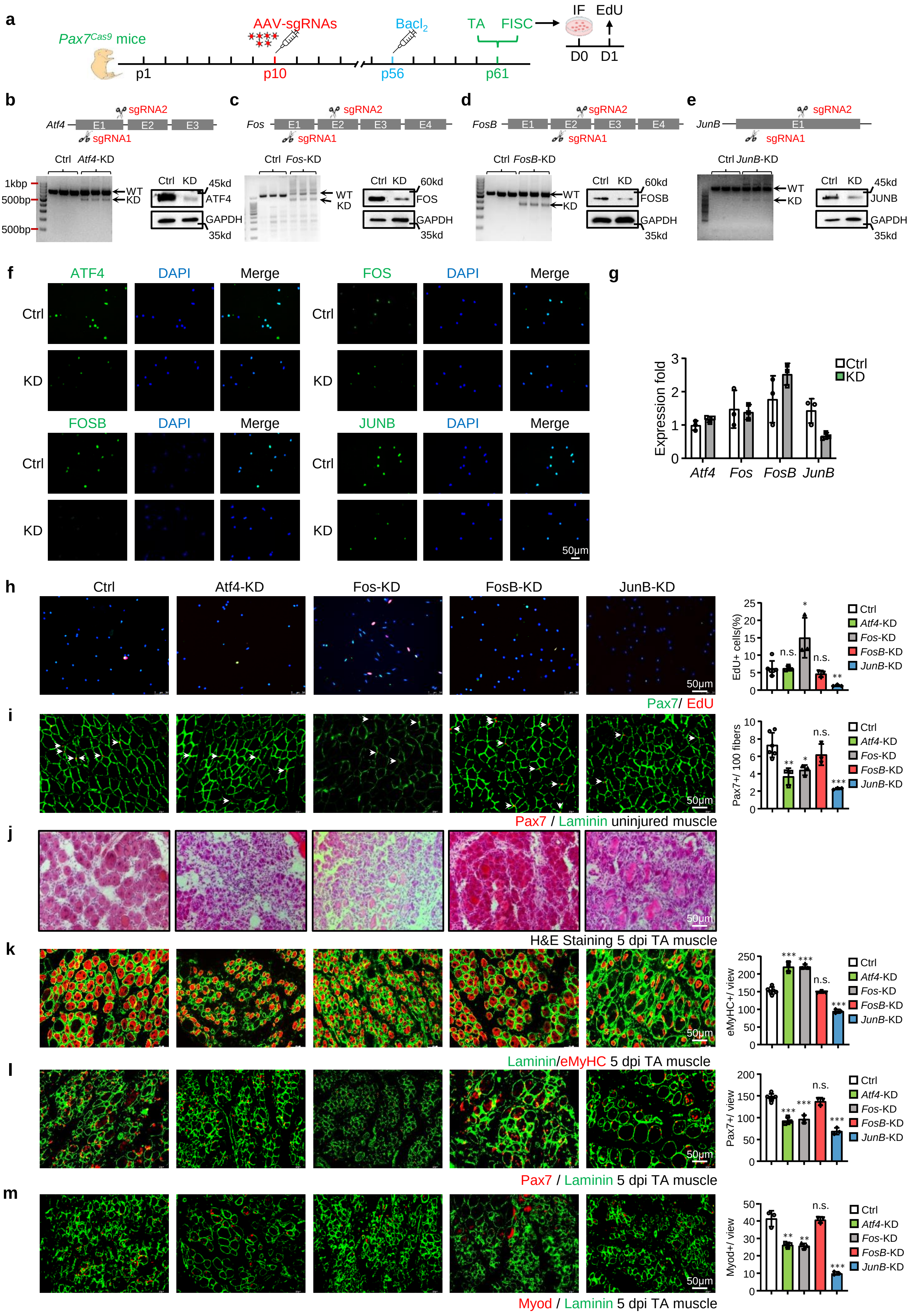

a
Pax7Cas9 mice
AAV-sgRNAs
Bacl2
TA
FISC
IF
EdU
D0
D1
p1
p10
p56
p61
b
Atf4
sgRNA2
sgRNA1
E1
E2
E3
Ctrl
Atf4-KD
1kbp
500bp
WT
KD
Ctrl
KD
45kd
ATF4
GAPDH
35kd
c
Fos
sgRNA2
sgRNA1
E1
E2
E3
E4
Ctrl
Fos-KD
WT
KD
60kd
FOS
GAPDH
35kd
d
FosB
sgRNA2
sgRNA1
Ctrl
FosB-KD
WT
KD
60kd
FOSB
GAPDH
35kd
e
JunB
sgRNA2
sgRNA1
Ctrl
JunB-KD
WT
KD
45kd
JUNB
GAPDH
35kd
f
ATF4
DAPI
Merge
FOS
FOSB
JUNB
Ctrl
KD
50μm
g
Expression fold
Ctrl
KD
Atf4
Fos
FosB
JunB
h
Ctrl
Atf4-KD
Fos-KD
FosB-KD
JunB-KD
50μm
EdU+ cells(%)
n.s.
Pax7/ EdU
i
Pax7+/ 100 fibers
Pax7 / Laminin uninjured muscle
j
H&E Staining 5 dpi TA muscle
k
eMyHC+/ view
Laminin/eMyHC 5 dpi TA muscle
l
Pax7+/ view
Pax7 / Laminin 5 dpi TA muscle
m
Myod+/ view
Myod / Laminin 5 dpi TA muscle

Supplementary Figure 8. Screening of other functional AP-1 family members in SCs and regeneration. a Schematic outline of the experimental design for screening of functional AP-1 members. **b-e** Upper: Illustration of locations of sgRNAs designed for targeting locus. Lower left: Editing efficiency. WT(Wild-type fragments) and KD(Cas9 cleaved fragments) are indicated by arrows. Lower right: protein levels of the above targeted AP-1 were examined by Western blotting in FISCs. GAPDH was used as a loading control. **f** Protein levels of the above targets were examined by IF staining. **g** The RNA levels of *Atf4*, *Fos*, *FosB* and *JunB* in the above FISCs were examined by RT–qPCR. **h** EdU (red) and Pax7 (green) staining of SCs sorted from *Ctrl* or *Atf4*, *FosB*, *Fos* and *JunB*-KD mice and cultured for 24h. Scale bar: 50μm. P = 0.99, 0.010, 0.27 and 0.0061. **i** IF staining of Pax7 (red) and Laminin (green) on TA muscles from the above KD mice 56 days after AAV injection. Scale bar: 50μm. P = 0.0055, 0.013, 0.29 and 0.00057. **j** H&E staining of the TA muscles collected from the above KO mice at 5dpi. Scale bar: 50μm. **k** IF staining of eMyHC (red) and Laminin (green) on the TA muscles from the above KD mice collected at 5dpi. Scale bar: 50μm. P = 0.000056, 0.000014, 0.49 and 0.000036. **l** IF staining of Pax7 (red) and Laminin (green) on the TA muscles from the KD mice collected at 5dpi. Scale bar: 50μm. P = 0.000033, 0.000067, 0.14 and 0.0000026. **m** IF staining of MyoD (red) and Laminin (green) on the TA muscles collected from the above KD mice at 5dpi. Scale bar: 50μm. P = 0.0056, 0.0050, 0.82 and 0.00031. n = 3 mice per group (b-e, g). For Ctrl, n = 6 mice per group. For *Atf4*, *FosB*, *Fos* and *JunB*-KD, n = 3 mice per group. (h-m). All the bar graphs are presented as mean ± SD. Student's t-test (two-tailed unpaired) was used to calculate the statistical significance (h-i, k-m): $*p < 0.05$, $**p < 0.01$, $***p < 0.001$. n.s., no significance. Source data are provided as a Source Data file.
