# Supplementary material for: ATF3 induction prevents precocious activation of skeletal muscle stem cell by regulating H2B expression
Source: Nat Commun. 2023 Aug 17;14:4978. doi: 10.1038/s41467-023-40465-w (PMC10435463; doi:10.1038/s41467-023-40465-w)
Supplement: Supplementary file 3 — Description of Additional Supplementary Files [file 41467_2023_40465_MOESM3_ESM.pdf]

Description of Additional Supplementary Files

Supplementary Data 1: Transcriptomic profiling in *Atf3* iKO SCs.

Supplementary Data 2: Genome-wide profiling of ATF3 binding.

Supplementary Data 3: Transcriptomic profiling in *Atf3* cKO SCs.

Supplementary Data 4: CUT&RUN analysis of H2B binding in *Atf3* iKO SCs.

Supplementary Data 5: Information of oligonucleotides and primers used in the study.
