# Supplementary material for: ATF3 induction prevents precocious activation of skeletal muscle stem cell by regulating H2B expression
Source: Nat Commun. 2023 Aug 17;14:4978. doi: 10.1038/s41467-023-40465-w (PMC10435463; doi:10.1038/s41467-023-40465-w)
Supplement: Supplementary file 9 — Reporting Summary [file 41467_2023_40465_MOESM9_ESM.pdf]

nature portfolio

Corresponding author(s): Huating Wang

Last updated by author(s): Jul 5, 2023

Reporting Summary

Nature Portfolio wishes to improve the reproducibility of the work that we publish. This form provides structure for consistency and transparency in reporting. For further information on Nature Portfolio policies, see our Editorial Policies and the Editorial Policy Checklist.

Statistics

For all statistical analyses, confirm that the following items are present in the figure legend, table legend, main text, or Methods section.

n/a	Confirmed	
☐	☒	The exact sample size (*n*) for each experimental group/condition, given as a discrete number and unit of measurement
☐	☒	A statement on whether measurements were taken from distinct samples or whether the same sample was measured repeatedly
☐	☒	The statistical test(s) used AND whether they are one- or two-sided<br>*Only common tests should be described solely by name; describe more complex techniques in the Methods section.*
☒	☐	A description of all covariates tested
☒	☐	A description of any assumptions or corrections, such as tests of normality and adjustment for multiple comparisons
☐	☒	A full description of the statistical parameters including central tendency (e.g. means) or other basic estimates (e.g. regression coefficient) AND variation (e.g. standard deviation) or associated estimates of uncertainty (e.g. confidence intervals)
☐	☒	For null hypothesis testing, the test statistic (e.g. *F*, *t*, *r*) with confidence intervals, effect sizes, degrees of freedom and *P* value noted<br>*Give P values as exact values whenever suitable.*
☒	☐	For Bayesian analysis, information on the choice of priors and Markov chain Monte Carlo settings
☒	☐	For hierarchical and complex designs, identification of the appropriate level for tests and full reporting of outcomes
☐	☒	Estimates of effect sizes (e.g. Cohen's *d*, Pearson's *r*), indicating how they were calculated

Our web collection on statistics for biologists contains articles on many of the points above.

Software and code

Policy information about availability of computer code

Data collection

qRT-PCR was conducted using the Light Cycler 480 Real-Time PCR System (Roche Applied Science);
Microscopy pictures were acquired with Leica microscope system DM600B.
BD FACSVerse flow cytometer, BD FACSAria Fusion Cell Sorter and BD FACSDiva (Version 8.0.1, BD Biosciences) were used for the acquisition of flow cytometry data.

Data analysis

Excel version 2208 Build 16.0.15601.20676 and GraphPad Prism version 8.0 was used for data analysis.
Leica LAS AF software (iLA.AF2.6.3) was used for the analysis of images from Leica microscope.
ImageJ 1.50 (National Institutes of Health) was used for quantification of Western blot band intensities.
WinMDI 2.8.was used to analyze flow cytometry data.
The analysis associated with RNA-seq was conducted mainly using custom code. Basic NGS-processing software including TopHat2 (v2.1.1), Cufflinks (v2.2.1), Bowtie2 (v2.4.4) were used. Differentially expressed genes (DEGs) between samples were identified by the change of expression levels using a threshold of log2FoldChange > 2. ClusterProfiler (v4.2.2) was used for the Gene Ontology (GO) analysis with Entrez gene IDs converted from DAVID tool as inputs. The adjusted P or P values were reported with the GO terms.
As for ATF3 ChIP-seq, Basic NGS-processing software including Bowtie2 2.4.4) was used, and only non-redundant reads were kept. The protein DNA-binding peaks (sites) were identified using MACS2 (v 2.2.7.1) with input (IgG) sample as the background. During the peak calling, the P-value cutoff was set to 0.001 for ATF3 ChIP-Seq experiment.
Additionally, CUT&RUN analysis, Basic NGS-processing software including Bowtie2 (v2.4.4) was used, and only non-redundant reads were kept. For the analysis of genome-wide differential H2B enrichment, we calculated the mean signal for H2B within each bin with 10 kb using the function "multiBigwigSummary bins" in deeptools (v3.5.1). Bins with average signal lower than 1 were removed. A threshold of 1.5-fold change was used to classify changed or unchanged bins between the ATF3-iKO samples and Ctrl samples. Only bins shared in at least 2 out of 3 replicates were considered as high-confidence bins. To analyze the differential H2B enrichment, we compared the signals on promoter and

gene body between ATF3-iKO and Ctrl samples with the Mann-Whitney U test. P-value < 0.05 and fold change > 1.5 were used to define significantly different. Only genes (including promoter or gene body) found in at least 2 out of 3 replicates were considered as high-confidence.

For manuscripts utilizing custom algorithms or software that are central to the research but not yet described in published literature, software must be made available to editors and reviewers. We strongly encourage code deposition in a community repository (e.g. GitHub). See the Nature Portfolio guidelines for submitting code & software for further information.

Data

Policy information about availability of data

All manuscripts must include a data availability statement. This statement should provide the following information, where applicable:

- Accession codes, unique identifiers, or web links for publicly available datasets
- A description of any restrictions on data availability
- For clinical datasets or third party data, please ensure that the statement adheres to our policy

RNA-Seq data of freshly isolated satellite cell from Ctrl and iKO or Ctrl and cKO mice, ATF3 ChIP-Seq and H2B CUT&RUN data generated in this study have been deposited in Gene Expression Omnibus (GEO) database under the accession codes GSE205170, GSE205314, GSE205324 and GSE205548.
Fig. 1c-e, 2c-k, 2m-n, 3a-d, 3f-k, 3o-q, 4b-g, 4i, 5b-g, 5i-n, 6k, 7b, 7e-g, 8a, 8c-f, 8h, Supplementary Fig. 1a, 2c-h, 3c, 3f-h, 4e-k, 4m, 4n, 5a-c, 6b, 6h, 7b-e, 7g-i, 7k-m contain associated raw data. All other data supporting the findings of this study are available from the corresponding author on reasonable request.

Research involving human participants, their data, or biological material

Policy information about studies with human participants or human data. See also policy information about sex, gender (identity/presentation), and sexual orientation and race, ethnicity and racism.

Reporting on sex and gender	*Use the terms sex (biological attribute) and gender (shaped by social and cultural circumstances) carefully in order to avoid confusing both terms. Indicate if findings apply to only one sex or gender; describe whether sex and gender were considered in study design; whether sex and/or gender was determined based on self-reporting or assigned and methods used. Provide in the source data disaggregated sex and gender data, where this information has been collected, and if consent has been obtained for sharing of individual-level data; provide overall numbers in this Reporting Summary. Please state if this information has not been collected. Report sex- and gender-based analyses where performed, justify reasons for lack of sex- and gender-based analysis.*
Reporting on race, ethnicity, or other socially relevant groupings	*Please specify the socially constructed or socially relevant categorization variable(s) used in your manuscript and explain why they were used. Please note that such variables should not be used as proxies for other socially constructed/relevant variables (for example, race or ethnicity should not be used as a proxy for socioeconomic status). Provide clear definitions of the relevant terms used, how they were provided (by the participants/respondents, the researchers, or third parties), and the method(s) used to classify people into the different categories (e.g. self-report, census or administrative data, social media data, etc.) Please provide details about how you controlled for confounding variables in your analyses.*
Population characteristics	*Describe the covariate-relevant population characteristics of the human research participants (e.g. age, genotypic information, past and current diagnosis and treatment categories). If you filled out the behavioural & social sciences study design questions and have nothing to add here, write "See above."*
Recruitment	*Describe how participants were recruited. Outline any potential self-selection bias or other biases that may be present and how these are likely to impact results.*
Ethics oversight	*Identify the organization(s) that approved the study protocol.*

Note that full information on the approval of the study protocol must also be provided in the manuscript.

Field-specific reporting

Please select the one below that is the best fit for your research. If you are not sure, read the appropriate sections before making your selection.

☒ Life sciences ☐ Behavioural & social sciences ☐ Ecological, evolutionary & environmental sciences

For a reference copy of the document with all sections, see nature.com/documents/nr-reporting-summary-flat.pdf

Life sciences study design

All studies must disclose on these points even when the disclosure is negative.

Sample size	At least three biological replicates per group (detailed n is indicated in the figure or figure legends) were collected to perform statistical testing. No statistical test was used to determine sample size. For ATF3 ChIP-seq , one biological replicate per group was used. For RNA-seq of muscle satellite cells, three samples per group were used. For H2B CUT&RUN-seq , three biological replicates per group were used.
Data exclusions	No data or samples were excluded from the analysis

Replication	All experimental data was repeated in multiple biological independent experiments as described in the legend, method and source data except for the ATF3 ChIP-seq, for which data of one experiment was shown.
Randomization	For all the animal experiments, we used the the same age and sex of Control and iKO or cKO mice from the same litter whenever possible. For cell experiments, we randomly counted multiple fields or cells per group for calculation.
Blinding	For immunofluorescence data collection , we performed the experiments in a blinded way. We randomly counted multiple fields per group and calculated the number of positively stained cells per field. For immunofluorescence intensity quantification, we were blinded to cell allocation during data analysis. RNA analyses using qRT-PCR and protein analyses using Western blotting were not performed blinded but by sample order.

Reporting for specific materials, systems and methods

We require information from authors about some types of materials, experimental systems and methods used in many studies. Here, indicate whether each material, system or method listed is relevant to your study. If you are not sure if a list item applies to your research, read the appropriate section before selecting a response.

Materials & experimental systems

n/a	Involved in the study
☐	☒ Antibodies
☐	☒ Eukaryotic cell lines
☒	☐ Palaeontology and archaeology
☐	☒ Animals and other organisms
☒	☐ Clinical data
☒	☐ Dual use research of concern
☒	☐ Plants

Methods

n/a	Involved in the study
☐	☒ ChIP-seq
☐	☒ Flow cytometry
☒	☐ MRI-based neuroimaging

Antibodies

Antibodies used	ATF3 (Santa Cruz Biotechnology, c-188x; 1:5,000), H2A (Abcam, ab177308; 1: 2,500), H2B (Abcam, ab1790; 1: 2,500), H3 (Santa Cruz Biotechnology, sc-8654; 1:4,000), H4 (Abcam, ab177840; 1:2,000), α-tubulin (Santa Cruz Biotechnology, sc-23948; 1:5,000) and GAPDH (Sigma-Aldrich, G9545-100ULSanta Cruz Biotechnology;1:5000) were used for Western blot.<br>PAX7 (Developmental Studies Hybridoma Bank, PAX7-S-1ML; 1:50), MyoD (Dako, M3512; 1: 1,000. Santa Cruz Biotechnology, sc-304x; 1:2,000), MyoG (Santa Cruz Biotechnology, sc-12732; 1:200), ATF3 (Santa Cruz Biotechnology, c-188x; 1:2,000), H2A (Abcam, ab177308; 1:1,000), H2B (Abcam, ab1790; 1:1,000), γ-H2AX (Biolegend, 613401; 1:200), ATF4 (Santa Cruz Biotechnology, 390063; 1:200), FOS (Santa Cruz Biotechnology, sc-8047; 1:200), FOSB (Santa Cruz Biotechnology, 398595; 1:200) and JUNB (Santa Cruz Biotechnology, 8051; 1:200) were used for immunofluorescence staining on cultured cells or myofibers.<br>PAX7 (Developmental Studies Hybridoma Bank, PAX7-S-1ML; 1:50), MyoD (Dako, M3512; 1:500), eMyHC (Developmental Studies Hybridoma Bank, F1.652; 1:200) Laminin (Sigma, L9393-100UL; 1:800) biotin-conjugated anti-mouse IgG (1:500 in 4% BBBSA, Jackson, 115-065-205) and Cy3-Streptavidin (1:1250 in 4% BBBSA, Jackson, 016-160-084)were used for immunofluorescence staining on sections.<br>10 µg of antibodies against ATF3 (Santa Cruz Biotechnology, c188-x), or normal mouse IgG (Santa Cruz Biotechnology, sc-2025) was used for immunoprecipitation.<br>5µg of H2B antibody (Abcam, ab1790) was used for CUT&RUN assay.
Validation	All antibodies used are commercially available and the applications have been tested by the manufacturers with the validation information for application and antigen specificity being provided in the respective data sheets from the manufacturers or on the manufacturers' websites.

Eukaryotic cell lines

Policy information about cell lines and Sex and Gender in Research

Cell line source(s)	Mouse C2C12 myoblast cells (CRL-1772) and 293T (CRL-3216) cells were obtained from American Type Culture Collection (ATCC).
Authentication	Cell lines were procured from commercial source and therefore, not authenticated.
Mycoplasma contamination	All cell lines were tested as negative for mycoplasma contamination.
Commonly misidentified lines (See ICLAC register)	No commonly misidentified lines were used in this study.

Animals and other research organisms

Policy information about studies involving animals; ARRIVE guidelines recommended for reporting animal research, and Sex and Gender in Research

Laboratory animals	The Tg: Pax7-nGFP mouse strains47, Pax7CreER (Pax7tm1(cre/ERT2)Gaka)89; ROSAEYFP reporter mice and Pax7Cre (Pax7tm1(cre)Mrc)58; ROSAEYFP reporter mice were kindly provided by Dr. Zhenguo WU (Hong Kong University of Science and Technology). The Atf3 fl/fl mouse strain was kindly provided by Prof. Tsonwin HAI (Ohio State University, USA)56. The C57BL wildtype mice were purchased from LASEC (Laboratory Animal Services Centre) of CUHK. The Atf3 inducible conditional KO mice (Atf3 iKO) with EYFP reporter (Ctrl: Pax7CreER/+; ROSAEYFP/+; Atf3+/+, iKO: Pax7CreER/+; ROSAEYFP/+; Atf3fl/fl) were generated by crossing Pax7CreER; ROSAEYFP with Atf3fl/fl mice. The Atf3 conditional KO mice (Atf3 cKO) with EYFP reporter (Ctrl: Pax7Cre/+; ROSAEYFP/+; Atf3+/+, cKO: Pax7Cre/+; ROSAEYFP/+; Atf3fl/fl) were generated by crossing Pax7Cre; ROSAEYFP with Atf3fl/fl mice. To induce Cre-mediated Atf3 deletion, Tamoxifen (TMX) (T5648, Sigma) was injected intraperitoneally at 2 mg per 20 g body weight for consecutive 5 days. Primers used for genotyping are shown in Suppl. Table 5. All the mice strains used in this study are ~2 month old.
Wild animals	No wild animals were used in this study.
Reporting on sex	No reporting on sex in this study.
Field-collected samples	No field-collected samples were used in this study.
Ethics oversight	All animal handling procedures, protocols and experiments ethics approval was granted by the CUHK AEEC (Animal Experimentation Ethics Committee) under the Ref No. 16-166-MIS and 21-254-MIS.

Note that full information on the approval of the study protocol must also be provided in the manuscript.

Plants

Seed stocks	*Report on the source of all seed stocks or other plant material used. If applicable, state the seed stock centre and catalogue number. If plant specimens were collected from the field, describe the collection location, date and sampling procedures.*
Novel plant genotypes	*Describe the methods by which all novel plant genotypes were produced. This includes those generated by transgenic approaches, gene editing, chemical/radiation-based mutagenesis and hybridization. For transgenic lines, describe the transformation method, the number of independent lines analyzed and the generation upon which experiments were performed. For gene-edited lines, describe the editor used, the endogenous sequence targeted for editing, the targeting guide RNA sequence (if applicable) and how the editor was applied.*
Authentication	*Describe any authentication procedures for each seed stock used or novel genotype generated. Describe any experiments used to assess the effect of a mutation and, where applicable, how potential secondary effects (e.g. second site T-DNA insertions, mosiacism, off-target gene editing) were examined.*

ChIP-seq

Data deposition

☒ Confirm that both raw and final processed data have been deposited in a public database such as GEO.

☒ Confirm that you have deposited or provided access to graph files (e.g. BED files) for the called peaks.

Data access links<br>*May remain private before publication.*	https://www.ncbi.nlm.nih.gov/geo/query/acc.cgi?acc=GSE205314
Files in database submission	GSE205314_RAW.tar
Genome browser session<br>(e.g. UCSC)	https://data.cyverse.org/dav-anon/iplant/home/fengyang/ATF3.sorted.rmdup.RPGCnorm.bw<br>https://data.cyverse.org/dav-anon/iplant/home/fengyang/ATF3_IgG_ctrl.sorted.rmdup.RPGCnorm.bw

Methodology

Replicates	1 ATF ChIP-seq; 1 IgG ChIP
Sequencing depth	Total reads: 5738565, Uniquely mapping reads: 3515570,100bp of raw reads,paired reads
Antibodies	ATF3 (Santa Cruz Biotechnology, C-19X)
Peak calling parameters	python macs2 callpeak -t ATF3.sorted.bam -c GmCtl.sorted.rmdup.bam --outdir ./ -n ATF3 -f BAM -g mm -p 0.001
Data quality	2871 peaks were called under cutoff p value <0.001

Software	Basic NGS-processing software including Bowtie2 2.4.4) was used, and only non-redundant reads were kept. The protein DNA-binding peaks (sites) were identified using MACS2 (v 2.2.7.1) with input (IgG) sample as the background.

Flow Cytometry

Plots

Confirm that:

- [x] The axis labels state the marker and fluorochrome used (e.g. CD4-FITC).
- [x] The axis scales are clearly visible. Include numbers along axes only for bottom left plot of group (a 'group' is an analysis of identical markers).
- [x] All plots are contour plots with outliers or pseudocolor plots.
- [x] A numerical value for number of cells or percentage (with statistics) is provided.

Methodology

Sample preparation	Briefly, entire hindlimb muscles from mice were digested with collagenase II (LS004177, Worthington, 1000 units per 1ml) for 90 min at 37°C, the digested muscles were then washed in washing medium (Ham's F-10 medium (N6635, Sigma) containing 10% horse serum, heat-inactivated (HIHS, 26050088, Gibco, 1% P/S) before SCs were liberated by treating with Collagenase II (100 units per 1ml) and Dispase (17105-041, Gibco, 1.1 unit per 1ml) for 30 min. The suspensions were passed through a 20 G needle to release myofiber-associated SCs. Mononuclear cells were filtered with a 40-µm cell strainer and sorted by BD FACSAria IV (fluorescence-activated cell sorting) with the selection of the positive GFP fluorescence signal.
Instrument	BD FACSAria Fusion Cell Sorter and BD FASC Calibur
Software	WinMDI 2.8.and BD FACSDiva
Cell population abundance	Muscle satellite cells sorting: we stained isolated cells by FACS with Pax7 antibody which is the marker of satellite cells and found 98% cells were Pax7 positive.
Gating strategy	Muscle satellite cells sorting: gating strategy was done according to previously reported protocol to sort out the GFP/YFP positive population.

- [x] Tick this box to confirm that a figure exemplifying the gating strategy is provided in the Supplementary Information.
